# Toxicity bioassays with concentrated cell culture media—a methodology to overcome the chemical loss by conventional preparation of water samples

**DOI:** 10.1007/s11356-018-1656-4

**Published:** 2018-03-10

**Authors:** Frida Niss, Anna Kjerstine Rosenmai, Geeta Mandava, Stefan Örn, Agneta Oskarsson, Johan Lundqvist

**Affiliations:** Department of Biomedical Sciences and Veterinary Public Health, Swedish University of Agricultural Sciences, Box 7028, SE-750 07 Uppsala, Sweden

**Keywords:** In vitro assays, Estrogen receptor activity, Aryl hydrocarbon receptor activity, Oxidative stress, Nrf2

## Abstract

The use of in vitro bioassays for studies of toxic activity in environmental water samples is a rapidly expanding field of research. Cell-based bioassays can assess the total toxicity exerted by a water sample, regardless whether the toxicity is caused by a known or unknown agent or by a complex mixture of different agents. When using bioassays for environmental water samples, it is often necessary to concentrate the water samples before applying the sample. Commonly, water samples are concentrated 10–50 times. However, there is always a risk of losing compounds in the sample in such sample preparation. We have developed an alternative experimental design by preparing a concentrated cell culture medium which was then diluted in the environmental water sample to compose the final cell culture media for the in vitro assays. Water samples from five Swedish waste water treatment plants were analyzed for oxidative stress response, estrogen receptor (ER), and aryl hydrocarbon receptor (AhR) activity using this experimental design. We were able to detect responses equivalent to 8.8–11.3 ng/L TCCD for AhR activity and 0.4–0.9 ng/L 17β-estradiol for ER activity. We were unable to detect oxidative stress response in any of the studied water samples. In conclusion, we have developed an experimental design allowing us to examine environmental water samples in toxicity in vitro assays at a concentration factor close to 1, without the risk of losing known or unknown compounds during an extraction procedure.

**Electronic supplementary material:**

The online version of this article (10.1007/s11356-018-1656-4) contains supplementary material, which is available to authorized users.

## Introduction

The application of bioanalytical tools, such as in vitro bioassays, for toxicity studies in environmental water samples is a rapidly expanding field of research (Escher et al. 2012; Tang et al. 2014; Busch et al. 2016; Lillicrap et al. 2016; Mehinto et al. 2016; König et al. 2017; Kunz et al. 2017; Neale et al. 2017a, b; Tan and Schirmer 2017). Currently, a large part of the environmental monitoring efforts are based on chemical analysis of known environmental pollutants. Generally, this is considered a suitable method when monitoring known pollutants with well-defined metabolites. However, targeted chemical analysis alone has limitations, as it is not possible to detect unknown substances. Furthermore, chemical analysis is not necessarily linked to adverse outcome and thus ignore possible mixture effects. Based on these limitations, and the rapid development of in vitro bioassays, it has repeatedly been suggested that a combination of chemical characterization and toxicological profiling would be a more suitable way of performing environmental monitoring, for a better understanding of the effects of environmental pollutants (Escher and Leusch 2012; Escher et al. 2013, 2017; Malaj et al. 2014; Petrie et al. 2015; Ankley et al. 2016; Brack et al. 2016, 2017).

When using cell-based bioassays for environmental monitoring purposes, it is often necessary to concentrate the water samples before applying the sample to the in vitro assay, as the levels of environmental pollutants are relatively low and the sample needs to be diluted approximately 100 times in cell culture media during the assay. It is common to use concentration techniques that give a final concentration factor in the cell culture media of 10–50 times concentration as compared to the original water sample, and in some cases, a concentration factor of up to 400 (König et al. 2017; Neale et al. 2017a, b). However, when concentrating environmental water samples (e.g., by solid phase extraction or by liquid-liquid extraction), there is always a risk of losing known or unknown compounds in the sample. This risk is difficult to control for when working with environmental samples, given the fact that such a large part of the observed toxicity is caused by unknown compounds (Escher et al. 2013; König et al. 2017; Neale et al. 2017b).

In this study, we have used an alternative experimental design by preparing a concentrated cell culture medium which was then diluted in the environmental water sample to compose the final cell culture media for the in vitro assays. The samples were analyzed for estrogen receptor (ER) activity, aryl hydrocarbon receptor (AhR) activity, and oxidative stress response (Nrf2 activity).

## Materials and methods

### Water sample collection

Effluent water was collected as 500 mL grab samples from the outlet of five Swedish waste water treatment plants (WWTP). Each WWTP serve between 90,000 and 190,000 persons and receive mixed sewage from municipal and industrial sources. The water samples, four samples from each waste water treatment plant, were collected during April–October 2016 (Table 1). The samples were transported at 8 °C and reach the laboratory within 24 h after sampling. Immediately upon arrival the samples were sterile filtered using a vacuum flask with a filter with a pore size of 0.22 μm (Sarstedt) and then kept at − 20 °C until analyzed.

**Table 1:** Water sample IDs, sampling sites, and sampling dates

Sample ID	Waste water treatment plant	Sampling date
1	Plant A	April 4, 2016
2	Plant B	April 4, 2016
3	Plant C	April 4, 2016
4	Plant D	April 4, 2016
5	Plant E	April 4, 2016
6	Plant A	May 10, 2016
7	Plant B	May 10, 2016
8	Plant C	May 10, 2016
9	Plant D	May 10, 2016
10	Plant E	May 10, 2016
11	Plant A	June 13, 2016
12	Plant B	June 13, 2016
13	Plant C	June 13, 2016
14	Plant D	June 13, 2016
15	Plant E	June 13, 2016
6	Plant A	October 3, 2016
17	Plant B	October 3, 2016
18	Plant C	October 3, 2016
19	Plant D	October 3, 2016
20	Plant E	October 3, 2016

### Cell culture of HepG2 cells and VM7Luc4E2 cells

For this study, we used the human hepatoma cell line HepG2 and the human breast cancer MCF7 cells stably transfected with an estrogen receptor sensitive luciferase plasmid (VM7Luc4E2 cells). Details on culturing conditions and cell culture media used are presented in Supplementary Information (SI, section 1.1).

### Preparation of concentrated cell culture media

For the exposure experiments, a ten times concentrated cell culture medium was prepared using a DMEM powder medium (Sigma Life Science) dissolved in deionized water and sterile filtered. The 10× cell culture media were then diluted into 1× using the environmental water samples. The dilution was performed on the day of experiment and diluted cell culture media were not stored for subsequent experiments. Further, the following additives were added to the 1× cell culture medium for assays based on HepG2 cells: 3.7 g/L sodium bicarbonate (Sigma), 4.5 g/L glucose (Gibco), 4 mg/L pyridoxine monohydrochloride (Sigma), 0.11 g/L sodium pyruvate 100 mM (Sigma), 10% heat inactivated fetal bovine serum (Gibco), 2 mM L-glutamine (Lonza BioWhittaker) and an antibiotic–antimycotic solution with a final concentration of 100 U/ml penicillin, 100 μg/ml streptomycin, and 0.25 μg/ml amphotericin B (Gibco). For experiments in the VM7Luc4E2 cells, the following components were added to the medium: 4.5% dextran-charcoal treated fetal bovine serum (Thermo Scientific), 4 mM L-glutamine (Lonza), 45 U/ml penicillin (Lonza), and 4.5 μg/ml streptomycin (Lonza).

The final concentration factor of the environmental water sample in the bioassays was 0.72. A concentration factor below 1 indicates that the samples have been diluted.

### Cell viability test

To ensure that the bioassays were performed under conditions that were not inducing cytotoxicity in the cells, a cell viability test was performed for all water samples. Details on the cell viability testing for each cell line are presented in Supplementary Information (SI, section 1.2).

### Oxidative stress response (Nrf2 activity) and AhR activity assays in transiently transfected HepG2 cells

To study the oxidative stress response, HepG2 cells were transiently transfected with an Nrf2 sensitive luciferase plasmid. To assay AhR activity, HepG2 cells were transiently transfected with an AhR sensitive luciferase plasmid. Following transfection, the cells were exposed to the water samples and the luciferase activity was measured. The luciferase activity was expressed as fold change compared to the vehicle treated cells. Details on the transfection procedure, exposure time and luciferase activity measurement are presented in Supplementary Information (SI, section 1.3).

### Estrogen receptor activity assay in VM7Luc4E2 cells

Estrogen receptor activity was assayed using the VM7Luc4E2 cell line, stably transfected with an estrogen sensitive luciferase plasmid. Cells were exposed to the water samples and the luciferase activity was measured. The luciferase activity was expressed as fold change compared to the vehicle treated cells. Details on the exposure time and luciferase activity measurement are presented in Supplementary Information (SI, section 1.4).

### Positive controls and cut-off values

The positive controls used were TCDD, sulforaphane and 17β-estradiol for the AhR, Nrf2 and ER assays, respectively. Further details are provided in Supplementary Information (SI, section 1.5).

The limit of detection (LOD) of the respective assays was based on the variation around the normalized vehicle control values (LOD = 1 + 3*SD). A cut-off value for positive response was then defined for each assay, as a value exceeding the LOD value. For the Nrf2 assay, the LOD was 1.7 and the cut-off value was set to 2. For ER, the LOD was 1.2 and the cut-off was defined as 1.5. Finally, for AhR, the LOD was 1.7 and the cut-off value was set to 2.

Positive controls for the AhR and ER assays were modeled by a four parameter sigmoidal curve fit. The estimated EC50, Hill slope, and maximum response were used to convert water sample responses into equivalent concentrations of TCDD and E2 by inserting water sample responses in the following equation:

$$ \log \left(\mathrm{concentration}\right)=\log \left({\mathrm{EC}}_{50}\right)-\frac{\log \left(\frac{\mathrm{top}-\mathrm{response}}{\mathrm{response}-\mathrm{bottom}}\right)}{\mathrm{hillslope}} $$

TCDD and E2 equivalent concentrations were only calculated for samples above the determined cut-off value, and the calculated equivalent values were corrected for the dilution of the water samples. For AhR and ER, the bioactivity of the positive controls were analyzed both in cell cultures with concentrated cell culture media and in cells cultured under standard conditions (described in SI, section 1.1). EC50 values for these experiments were calculated via modeling by a four parameter sigmoidal curve fit in GraphPad Prism 7. The results from these experiments are presented in Supplementary Information (SI, section 2).

## Results

### Cell viability

**Fig. 1:**
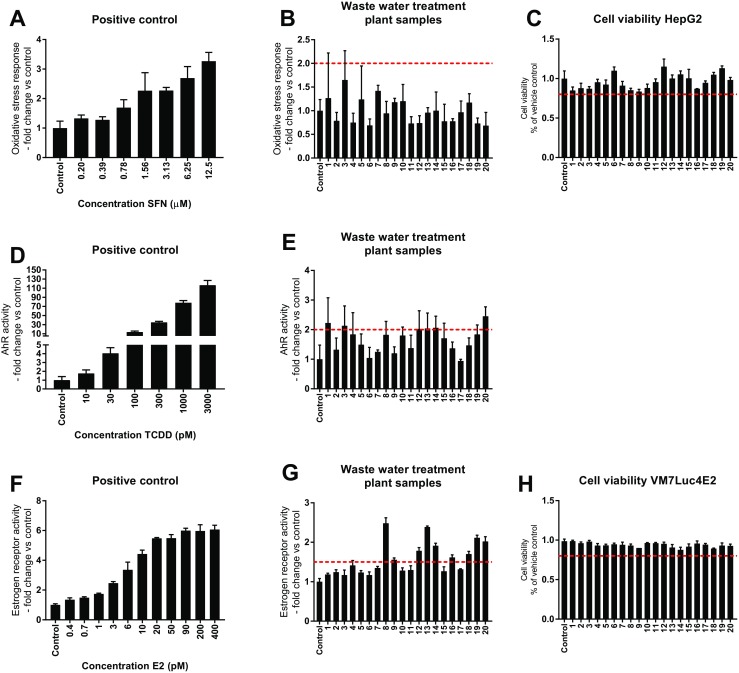
Toxicity bioassays with positive controls and waste water treatment plant outlet samples. Oxidative stress response for positive control (**a**) and water samples (**b**). Cell viability for HepG2 cells after exposure to water samples (**c**). AhR activity for positive control (**d**) and water samples (**e**). ER activity for positive control (**f**) and water samples (**g**). Cell viability for VM7Luc4E2 after exposure to water samples (**h**). Cells were exposed to positive controls or concentrated water samples for 24 h prior to measurement of luciferase activity (mean ± standard deviation, *n* = 4–8). Dashed lines in panels (**b**), (**e**), and (**g**) represent cut-off values defined in “Positive controls and cut-off values” section. Dashed lines in panels (**c**) and (**h**) represent threshold for cytotoxicity. For water sample numbering, refer to Table 1

To ensure that the experiments were conducted under non-cytotoxic conditions, the water samples were analyzed for their effect on the cell viability of HepG2 cells and VM7Luc4E2 cells. We found that no sample exerted a general cytotoxicity, defined as a cell viability below 80%, as compared to the pure water control, in neither the HepG2 cells, nor in the VM7Luc4E2 cells (Fig. 1c, h).

### Oxidative stress response

The oxidative stress response was assayed in HepG2 cells transiently transfected with an Nrf2 sensitive luciferase plasmid. For the positive control sulforaphane, we were able to detect an increased Nrf2 response in the low micromolar range (Fig. 1a). However, none of the waste water treatment plant outlet samples exerted any oxidative stress, defined as ≥ 2-fold increase in Nrf2 activity compared to vehicle control, in this experimental system (Fig. 1b).

### Aryl hydrocarbon receptor activation

The aryl hydrocarbon receptor activity of the water samples was assayed in HepG2 cells transfected with an AhR sensitive luciferase plasmid. The known AhR ligand TCDD increased the receptor activity in a dose-dependent manner (Fig. 1d). Of the investigated environmental water samples, we found that six out of 20 analyzed samples activated the AhR, defined as ≥ 2-fold increase in the AhR activity compared to the vehicle control (Fig. 1e). For the water samples that showed an AhR activity above the cut-off value, we calculated the TCDD equivalents (EQ). The TCDD EQs for samples 1, 3, 12, 13, 14, and 20 were 10.0, 9.5, 8.8, 9.0, 9.1, and 11.3 ng/L, respectively, corresponding to a TCDD EQ of 30–35 pM.

### Estrogen receptor activation

The estrogen receptor activity of the water samples was assayed in the VM7Luc4E2 cell line. The known estrogen receptor ligand 17β-estradiol increased the receptor activity in a dose-dependent manner (Fig. 1f). We found that nine out of the 20 analyzed water samples activated the estrogen receptor, defined as ≥ 1.5-fold increase in the ER activity compared to the vehicle control (Fig. 1g). For the samples with an ER activity above the cut-off value, we calculated the estradiol equivalents (EQ). The estradiol EQs for samples 8, 9, 12, 13, 14, 16, 18, 19, and 20 were 0.9, 0.4, 0.5, 0.9, 0.5, 0.4, 0.4, 0.6, and 0.6 ng/L, respectively, corresponding to an estradiol EQ of 1.3–3.5 pM.

## Discussion

Sample preparation to increase the concentration factor is always associated with a risk of losing compounds in the sample. This risk is especially difficult to control for in regard to the risk of losing unknown compounds. It has repeatedly been reported that the observed toxic effects of water samples in toxicity bioassays to a large extend is caused by unknown compounds. For example, Neale et al. (2017b) performed an integrated toxicological and chemical characterization of wastewater effluent water samples, analyzing the samples in 13 toxicity bioassays and for 405 chemical compounds. For AhR activity, the analyzed compounds could explain 0–30% of the observed toxic effect, for oxidative stress response (Nrf2 activity) the analyzed compounds explained 0.2–2% of the observed toxicity, and for ER activity the analyzed compounds could only explain 0.1–0.3% of the observed effect. These results are in line with other publications in this field (Escher et al. 2013; König et al. 2017). These findings strongly suggest that the total toxicity of an environmental water sample is exerted by a combination of known compounds and unknown compounds, of either natural or anthropogenic origin.

Sample enrichment methods are useful when analyzing water samples in bioassays, but also associated with a risk of losing known or unknown compounds in the sample. With the experimental design presented in this study, we minimize the risk of losing unknown compounds in the sample, while keeping the concentration factor at a level where we can detect toxic effects. In our assays, the final concentration factor was 0.72. Analyzing water samples with a final concentration factor of 0.72 is associated with a risk of not being able to detect bioactivity due to low levels of bioactive compounds in the sample, especially if analyzing samples from areas with low levels of bioactive compounds (i.e., areas with very low pollution). On the other hand, the polluted areas are more relevant to monitor. Therefore, we hypothesize that the methodology described in this study will be most useful for water samples from contaminated areas where relatively high levels of bioactive compounds can be expected. For samples from more pristine areas, sample preparation to increase the concentration factor might be necessary, although such sample preparation methods are associated with the risk of losing bioactive compounds during sample preparation. In addition, using concentrated culture media makes the procedure simpler, faster and cheaper compared to SPE.

We used the established experimental design to test the toxicity of effluent water samples from five Swedish wastewater treatment plants. We could observe significant bioactivity by water samples, below the actual concentrations present in the environment (a concentration factor of 0.72 means that any compound in the sample is present in a concentration 28% below the actual concentration in the initial water sample). The observed TCDD EQs in this study ranged from 8.8–11.3 ng/L. Previous studies have reported a wide range of TCDD EQs for waste water treatment plant outlets, from the low pg/L range to hundreds of ng/L. (Ma et al. 2005; Mahjoub et al. 2009; Dagnino et al. 2010; Macova et al. 2010; Reungoat et al. 2010; Mahjoub et al. 2011). The estradiol EQs observed for water samples in this study was in the range 0.4–0.9 ng/L, which is lower or comparable to other studies of waste water treatment plant outlets (Dagnino et al. 2010; Omoruyi and Pohjanvirta 2015; Välitalo et al. 2017). The detected toxic responses show that this experimental design might be useful when using bioanalytical tools for water quality assessments of environmental water samples, especially for water samples from contaminated areas.

## Electronic supplementary material

ESM 1 (PDF 165 kb)
