# Supplementary material for: Toxicity bioassays with concentrated cell culture media—a methodology to overcome the chemical loss by conventional preparation of water samples
Source: Environ Sci Pollut Res Int. 2018 Mar 10;25(12):12183–8. doi: 10.1007/s11356-018-1656-4 (PMC5940719; doi:10.1007/s11356-018-1656-4)
Supplement: Supplementary file 1 — (PDF 165 kb) [file 11356_2018_1656_MOESM1_ESM.pdf]

Supplementary Information

Toxicity bioassays with concentrated cell culture media - a methodology to overcome the chemical loss by conventional preparation of water samples

Frida Niss, Anna Kjerstine Rosenmai, Geeta Mandava, Stefan Örn, Agneta Oskarsson and Johan Lundqvist

Department of Biomedical Sciences and Veterinary Public Health, Swedish University of Agricultural Sciences, Box 7028, SE-750 07 Uppsala, Sweden

1. Experimental procedures

1.1 Culture conditions for HepG2 cells and VM7Luc4E2 cells

The human hepatoma cell line HepG2 (ECACC number 85011430) was cultured in Dulbecco's Modified Eagle Medium (DMEM) with 4.5 g/L glucose (Lonza, BioWhittaker), supplemented with 10% heat inactivated fetal bovine serum (Gibco), 2 mM L-glutamine (Lonza BioWhittaker), and an antibiotic–antimycotic solution with a final concentration of 100 U/ml penicillin, 100 µg/ml streptomycin, and 0.25 µg/ml amphotericin B (Gibco). TrypLE Express without Phenol Red (Gibco) was used for subculturing the cells twice per week. Medium was exchanged every 2-3 days.

Human breast cancer MCF7 cells stably transfected with an estrogen receptor sensitive luciferase plasmid (VM7Luc4E2 cells) were routinely cultured in RPMI 1640 (Lonza) supplemented with 8% fetal bovine serum (FBS) (Gibco), 45 U/ml penicillin (Lonza), and 4.5 µg/ml streptomycin (Lonza).

All cells were cultured in a humidified environment at 37ºC and 5% CO_2.

1.2 Cell viability testing

To assay the cell viability in HepG2 cells, the capacity of the cells to reduce a tetrazolium compound (MTS) to the coloured formazan was analyzed. The reaction is dependent on mitochondrial function and the amount of formazan formed is directly proportional to the number of living cells in culture. HepG2 cells were seeded in a density of 20,000 cells per well in a 96 well plate. Medium was exchanged 48 hours after seeding and after an additional 24 h the cells were treated with exposure medium. As a control, the cells were treated with concentrated cell culture media diluted with deionized water. 24 h after initiation of exposure, the Celltiter 96® AQ_{ueous} One Solution Reagent (Promega) was added and the cell viability was then assayed in accordance with the manufacturer's instructions using a Wallac Victor^2 1420 microplate reader (PerkinElmer) and relative effects on cell viability was calculated in relation to the vehicle control.

For VM7Luc4E2 cells, the cell viability was analyzed using the CellTiter-Glo® Luminescent Cell Viability Assay (Promega). For this assay, cells were seeded in a density of 40,000 cells per well in a 96 well plate. The cells were incubated for 24 h and successively treated with exposure media for 24 hours. After the exposure, the cell viability assay substrate was added and the luminescence was measured in a Wallac Victor2 1420 microplate reader (PerkinElmer) and relative effects on cell viability was calculated in relation to the vehicle control.

1.3 Oxidative stress response (Nrf2 activity) and AhR activity assays in transiently transfected HepG2 cells

HepG2 cells were seeded in a density of 20,000 cells per well in a 96 well plate, using standard cell culture medium. The cells were incubated for 48 h before they were transfected with 30 ng/well of renilla plasmid and either a plasmid for Nrf2 activation or a plasmid for AhR activation. For the Nrf2 reporter gene assay the cells were transfected with 30 ng/well of a Nrf2 responsive luciferase plasmid (pGL4.37[luc2P/ARE/Hygro])(Promega) [1]. For the AhR reporter gene assay the cells were transfected with 30 ng/well of an AhR responsive luciferase plasmid (PGudLuc7.5)[2]. This plasmid was a kind gift from Professor Michael Denison, University of California at Davis. For all assays, the DNA was delivered in 10 µl of Opti-MEM® (1x) reduced serum medium (Gibco) with 0.3 µL Lipofectamine® 2000 Reagent (Invitrogen by Life Technologies) per well, as recommended by the manufacturer.

Following the transfection, the cells were incubated for 24 h and then exposed to water samples, control samples or positive controls. After an additional 24 h the Dual-Luciferase® Reporter 1000 Assay System (Promega) was performed essentially according to the manufacturer's protocol by removing exposure medium and lysing for 30 minutes at room temperature before addition of assay reagents. The luminescence was analyzed using an Infinite M1000 plate reader (Tecan). The luciferase activity was expressed as fold change compared to the control treated group.

1.4 Estrogen receptor activity assay in VM7Luc4E2 cells

Estrogen receptor activity was assayed using the VM7Luc4E2 cell line, a cell line described in OECD Test Guideline 457 [3]. In this study, the protocol was adapted for usage with concentrated cell culture medium. The cell line VM7Luc4E2 contains a stably transfected firefly luc reporter construct, controlled by estrogen response elements, which will detect substances with *in vitro* ER agonist activity. This cell line was a generous gift from Michael Denison, University of California at Davis [4, 5].

Cells were seeded in a 96 well plate with a density of 40,000 cells per well and left to incubate for 24 h before being exposed to an exposure media based on DMEM (described above). After 24 hours of exposure, the luciferase activity was assayed using the Luciferase Assay System (Promega). The luminescence was analyzed using an Infinite M1000 plate reader (Tecan). The luciferase activity was expressed as fold change compared to the control treated group.

1.5 Positive controls

For each assay, a positive control was included as a standard curve. In the AhR assay TCDD (SUPELCO, Sigma-Aldrich) was used as a positive control in concentrations from 10 to 3000 pM. In the Nrf2 assay sulforaphane (Sigma-Aldrich) was used as a positive control in concentrations from 0.2 to 6.25 µM. The positive controls were dissolved in ethanol or DMSO. For the estrogen receptor activation assay 17β-estradiol (Sigma) and methoxychlor (Fluka) were used as positive controls. The concentration range for 17β-estradiol was 0.4 to 400 pM and methoxychlor was used in a single concentration of 0.9 µM. The positive controls were added to cell culture media that had been prepared by dilution of concentrated cell culture media with deionized water. The final vehicle concentration never exceeded 0.2 % in the cell culture media.

2. Results

2.1 Effect of TCCD and E2 under different culturing conditions

For the AhR and ER assays, the positive controls were analyzed both in assays performed with concentrated cell culture media (described in section 2.3 of this study) and in assays performed under standard culturing conditions (described in SI, section 1.1). The bioactivity of the positive controls, presented as percent of maximum response, is presented in Figure SI1. EC50 was calculated as described in section 2.7. EC50 for TCDD was 0.9 nM when the assay was performed with concentrated cell culture medium and 0.8 nM when the assay was performed under standard culturing conditions. EC50 for E2 was 6 pM when the assay was performed with concentrated cell culture medium and 26 pM when the assay was performed under standard culturing conditions.

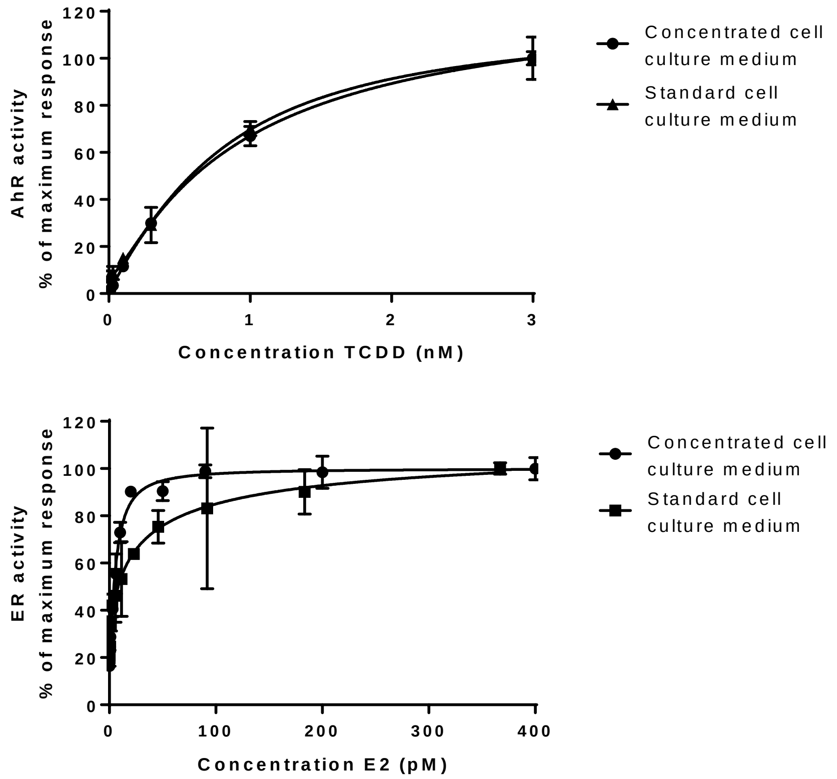

Figure SI1. Positive controls analyzed either with concentrated cell culture medium or under standard culturing conditions.

References

1. Lundqvist, J., B. Hellman, and A. Oskarsson, *Fungicide prochloraz induces oxidative stress and DNA damage in vitro.* Food Chem Toxicol, 2016. **91**: p. 36-41.

2. He, G., T. Tsutsumi, B. Zhao, D.S. Baston, J. Zhao, S. Heath-Pagliuso, and M.S. Denison, *Third-generation Ah receptor-responsive luciferase reporter plasmids: amplification of dioxin-responsive elements dramatically increases CALUX bioassay sensitivity and responsiveness.* Toxicol Sci, 2011. **123**(2): p. 511-22.
3. OECD, *Test No. 457: BG1Luc Estrogen Receptor Transactivation Test Method for Identifying Estrogen Receptor Agonists and Antagonists*. 2012: OECD Publishing.
4. Rogers, J.M. and M.S. Denison, *Recombinant cell bioassays for endocrine disruptors: development of a stably transfected human ovarian cell line for the detection of estrogenic and anti-estrogenic chemicals.* In Vitr Mol Toxicol, 2000. **13**(1): p. 67-82.
5. Brennan, J.C., A. Bassal, G. He, and M.S. Denison, *Development of a recombinant human ovarian (BG1) cell line containing estrogen receptor α and β for improved detection of estrogenic/antiestrogenic chemicals.* Environmental Toxicology and Chemistry, 2016. **35**(1): p. 91-100.
